# Supplementary material for: The Functional Epididymal Amyloid Cystatin‐Related Epididymal Spermatogenic (CRES) is a Component of the Mammalian Brain Extracellular Matrix
Source: J Neurosci Res. 2026 Jan 20;104(1):e70113. doi: 10.1002/jnr.70113 (PMC12818389; doi:10.1002/jnr.70113)
Supplement: Supplementary file 1 — Data S1: jnr70113‐sup‐0001‐DataS1.pdf. [file JNR-104-e70113-s002.pdf]

Supporting information

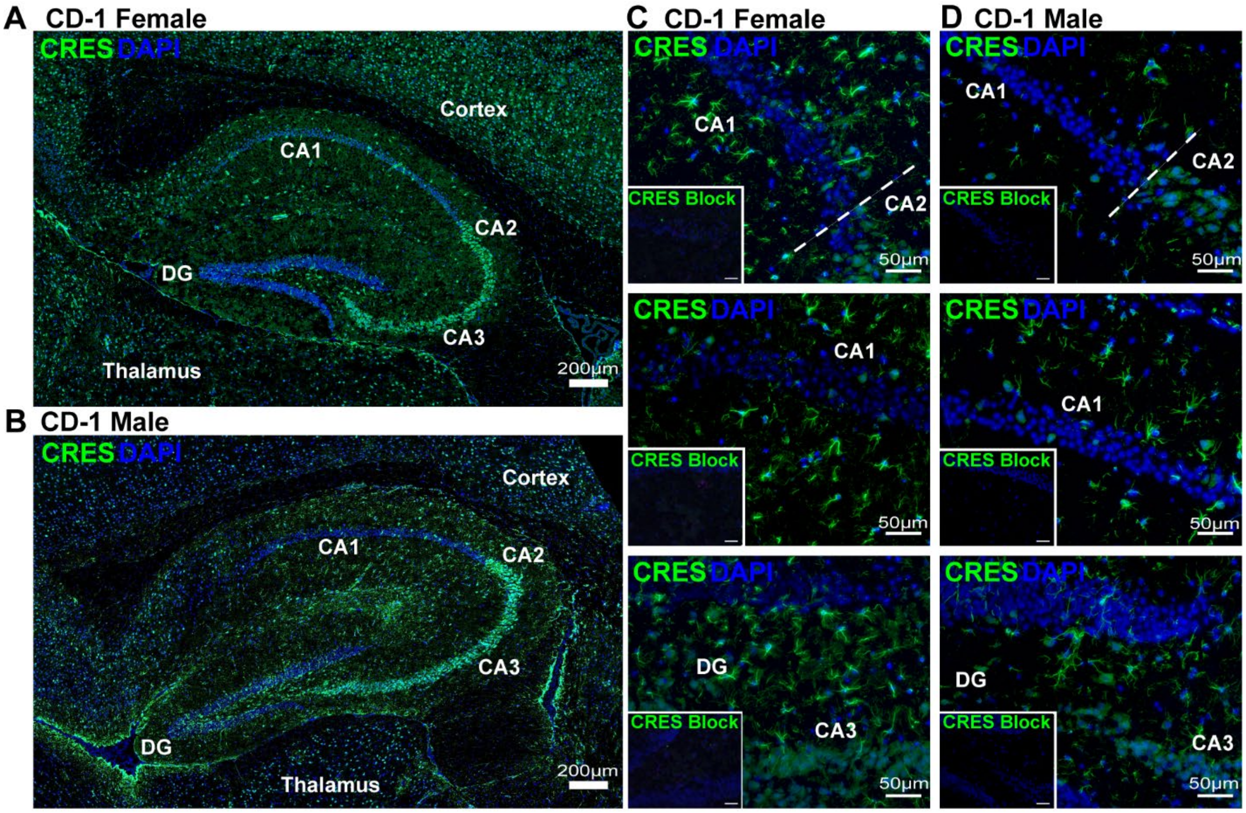

Figure S1. CRES localization in the mouse hippocampus is region-specific. CRES (green fluorescence) localization in the **A) C)** CD-1 female and **B), D)** CD-1 male mouse (~20 wks) hippocampus. CRES is present in CA3 and CA2 but not CA1 neurons or neurons in the dentate gyrus (DG). CRES immunoreactivity in the CA1 region and dentate gyrus (DG) is mostly in non-neuronal cells. Blue, DAPI staining of nuclei. Inset, CRES antibody preincubated with recombinant CRES protein (block) serve as a negative control. Scale bar, 50 µm.

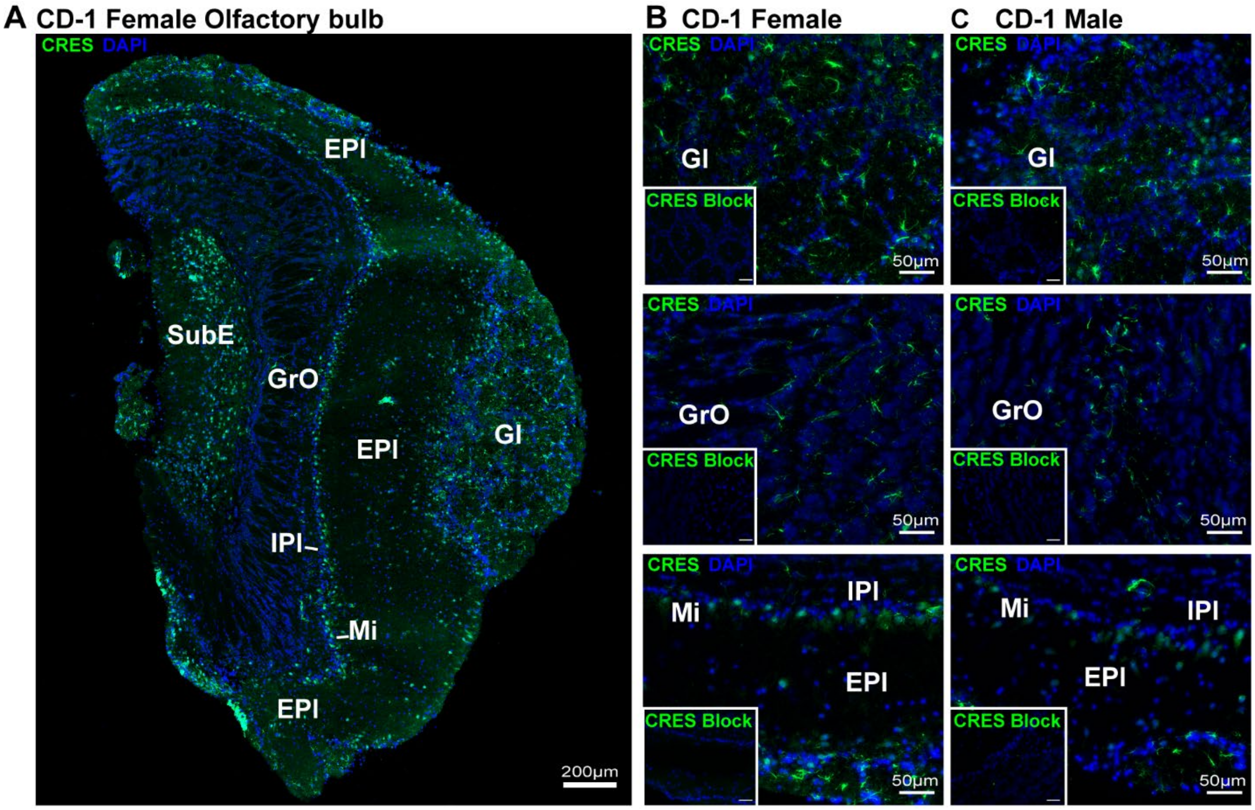

Figure S2. CRES localization in the mouse olfactory bulb. Immunofluorescence analysis was performed on cryopreserved mouse olfactory bulb (10 µm sagittal sections) from female and male CD-1 mice (~20 wks). **A)** CRES fluorescence (green) was present in distinct layers of the olfactory bulb. Blue, DAPI staining of nuclei. Scale bar, 200 µm. Magnified regions of **B)** female and **C)** male olfactory bulb revealed CRES fluorescence primarily in the glomerular layer (Gl), Mitral cell layer (Mi), and subependymal layer (SubE) with fewer positive cells in the granular cell layer (GrO). IPl, internal plexiform layer; EPl, external plexiform layer. Inset, CRES antibody preincubated with recombinant CRES protein (block) serve as a negative control. Scale bar, 50 µm.

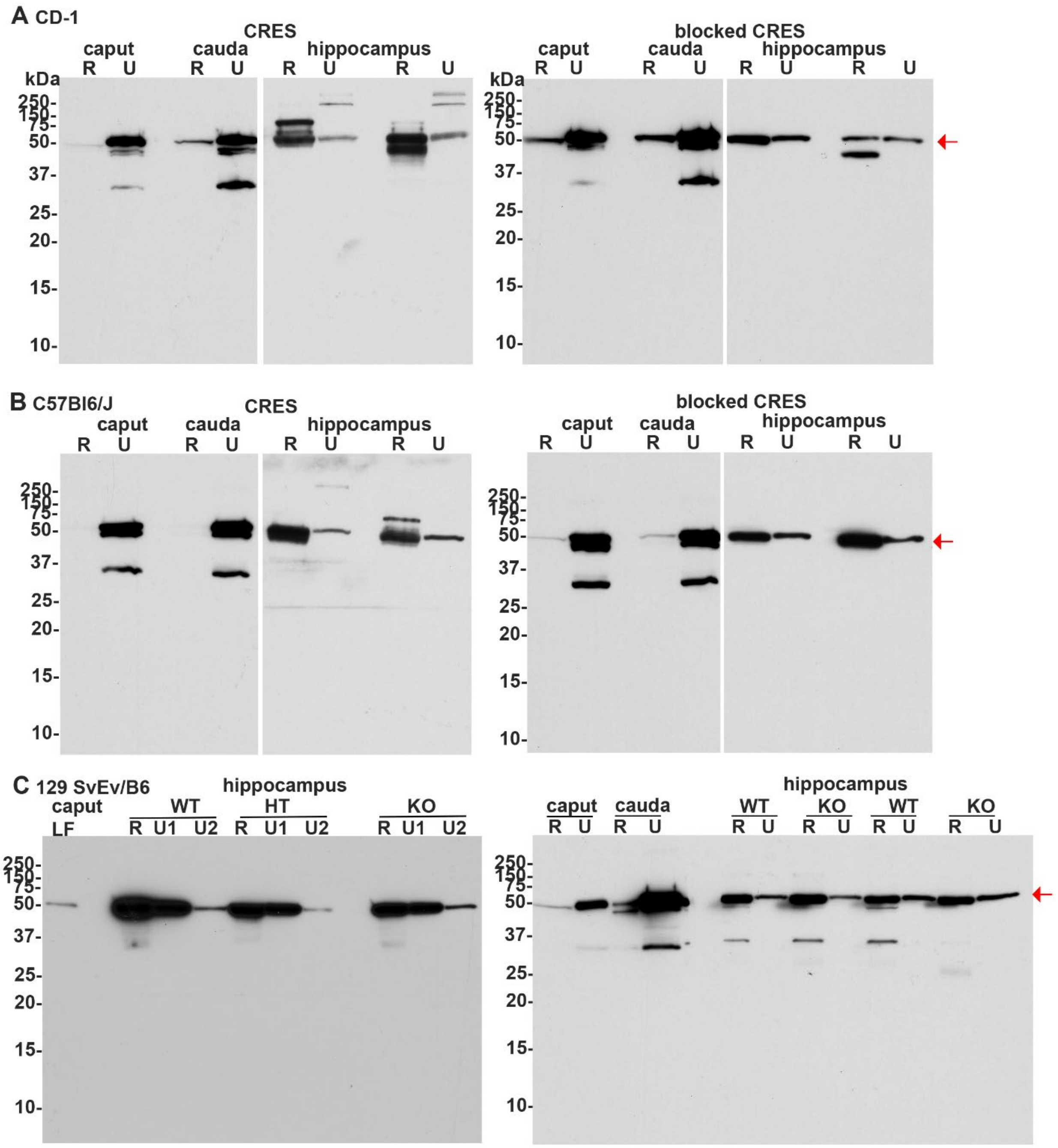

Figure S3. Full-length Western blots of α-tubulin (red arrows) in mouse RIPA (R) and urea (U) extracts from caput and cauda epididymis and hippocampus from three mouse strains. Cropped images are shown in Figure 5.

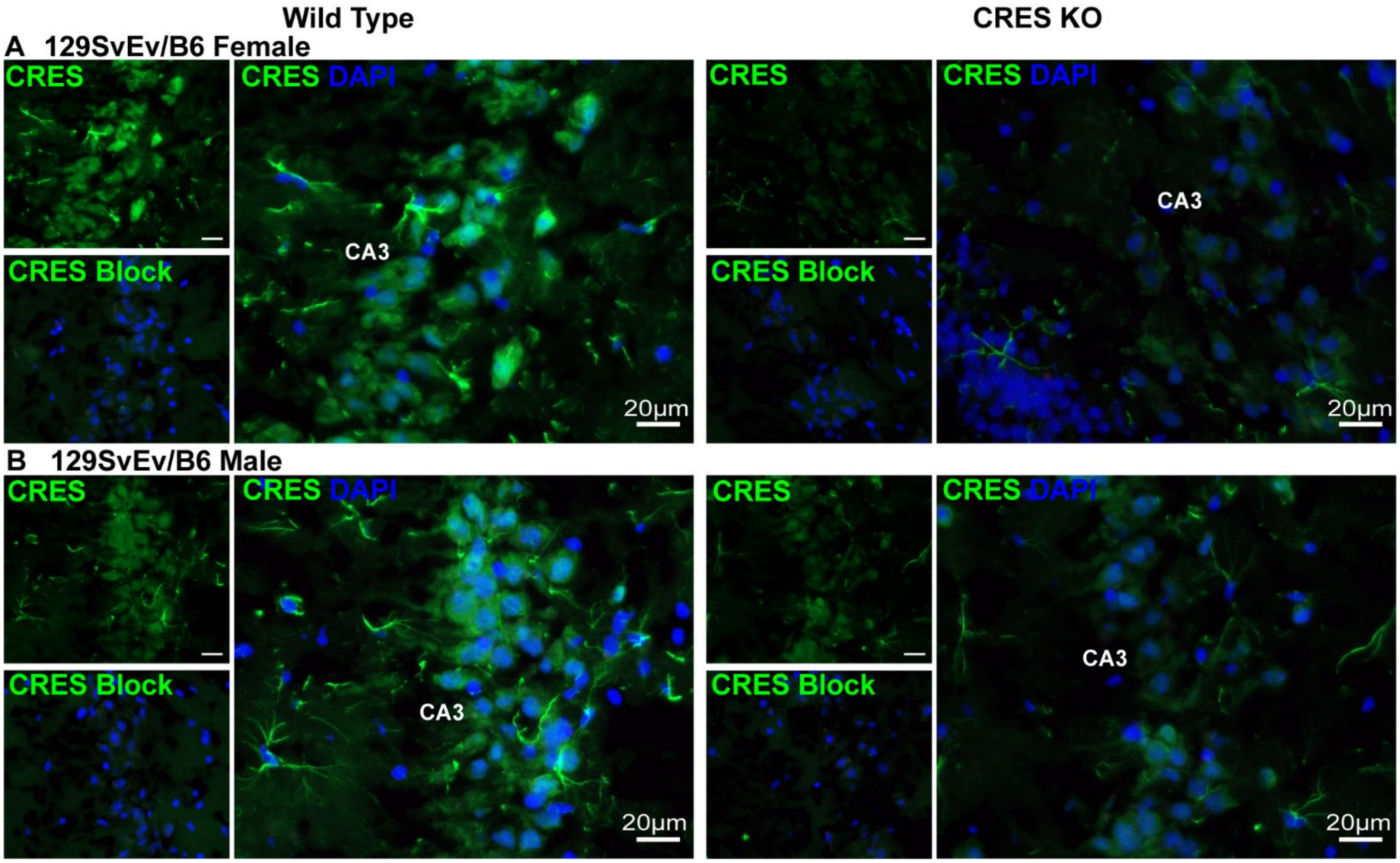

Figure S4. Female and male 129SvEv/B6 CRES KO mice show a loss of CRES in the hippocampus compared to WT mice. Immunofluorescence analysis showed CRES (green fluorescence) markedly decreased in hippocampal neurons and astrocytes from CRES KO female and male mice compared to wildtype (WT) controls. The CA3 region is shown. Blue fluorescence, DAPI staining of nuclei. The faint remaining fluorescence may represent a cross-reactivity of CRES antibody with other CRES subgroup members. CRES antibody blocked with CRES protein was used as negative control. Scale bar, 20 μm.

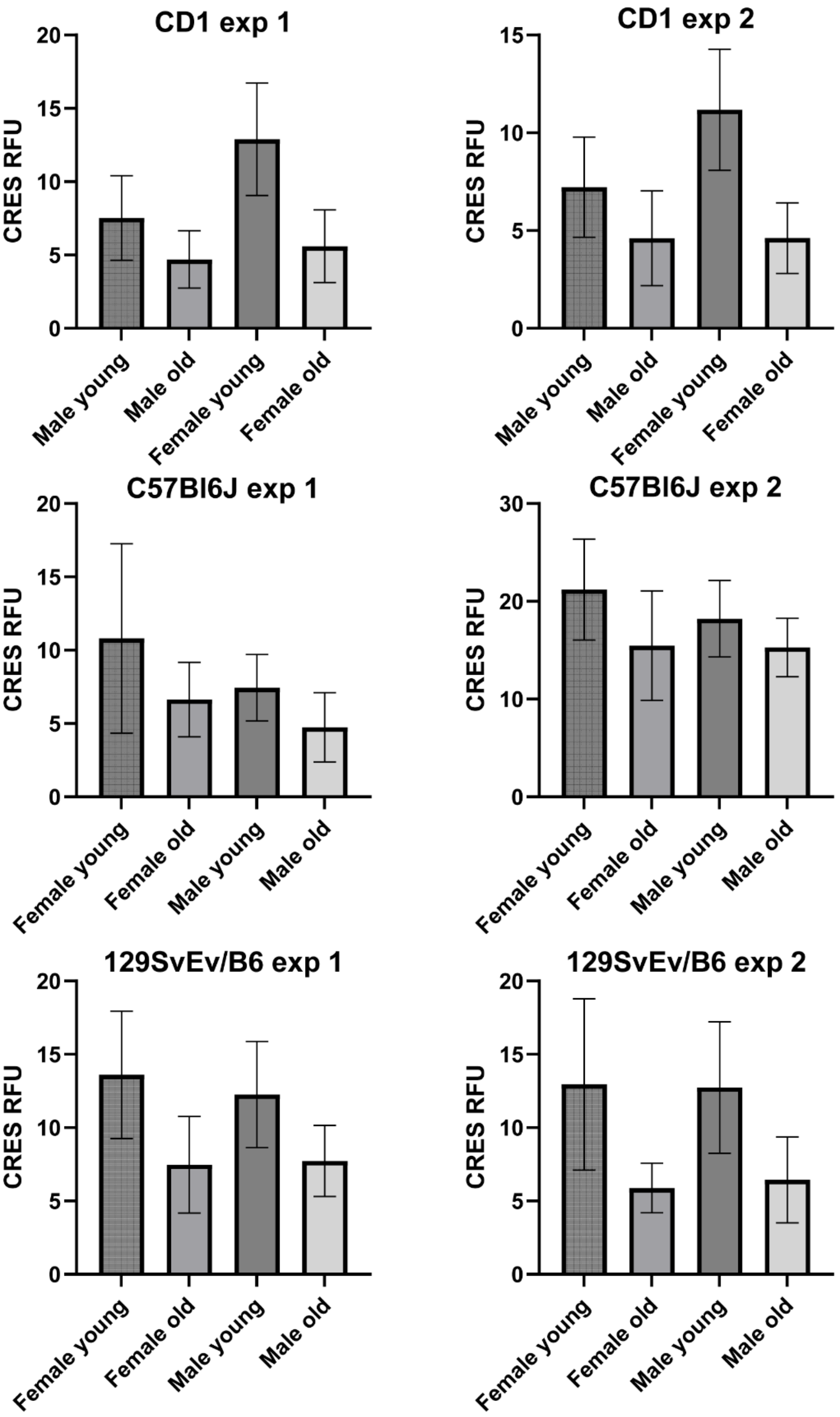

Figure S5. CRES fluorescence intensity levels in hippocampal astrocytes from three strains of young and old mice. Data shown represent the mean $\pm$ SD of the CRES relative fluorescence intensity (RFU) from individual astrocytes from young (~18-20 wks) and old (~60-67 wks) female and male CD-1, C7Bl6/J, and 129 SvEv/B6 mouse strains from two separate experiments.

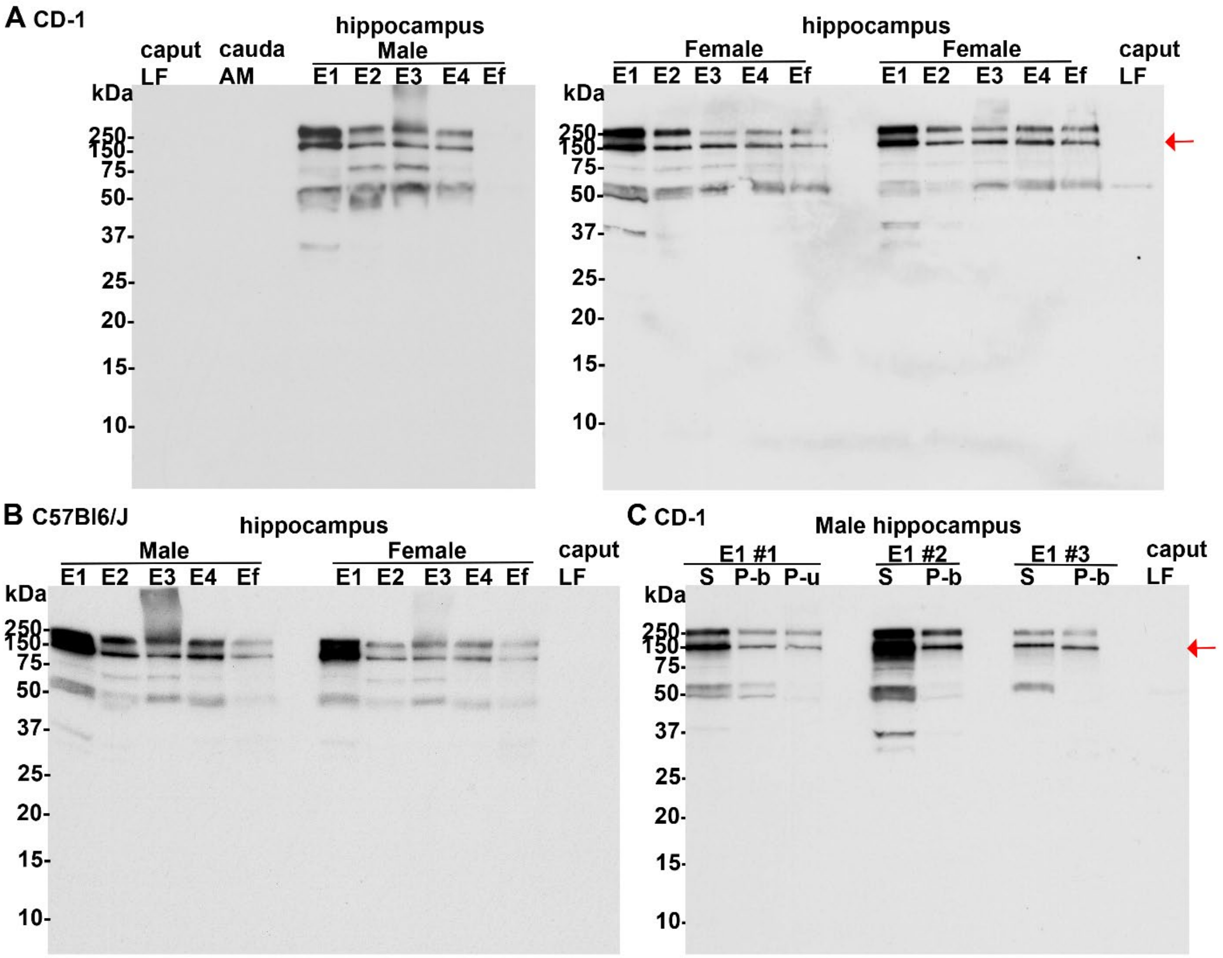

Figure S6. Full-length Western blots of brevican (red arrows) in hippocampal extracts from CD-1 and C57Bl6/J female and male mice. Higher molecular weight forms may represent differentially glycosylated and/or membrane-associated brevican while lower molecular weight proteins (80 kDa and 50 kDa) are likely processed forms. The E1#3 supernatant (S) sample in **C)** was underloaded. Cropped images are shown in Figure 10.

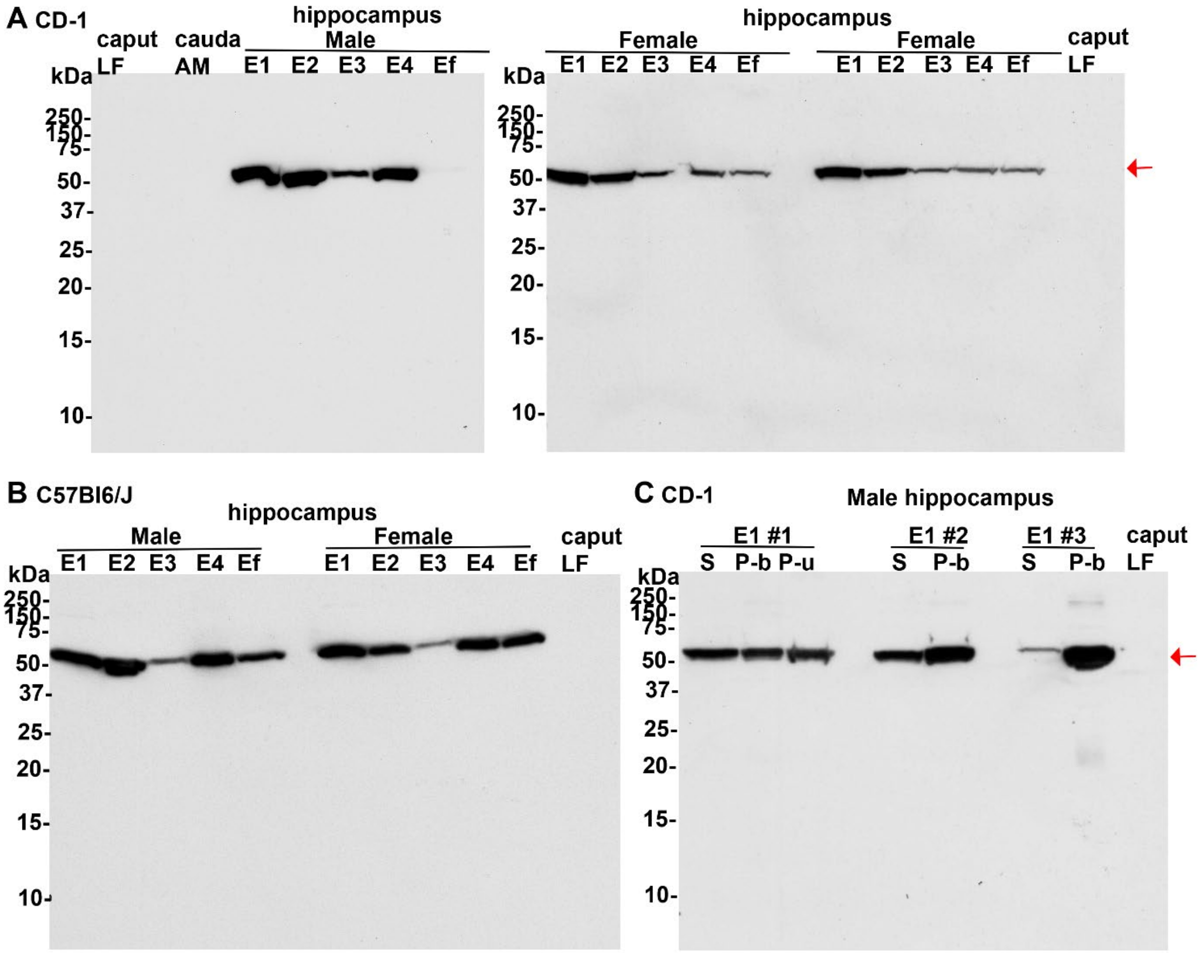

Figure S7. Full-length Western blots of β-III tubulin (red arrows) in hippocampal extracts from CD-1 and C57Bl6/J female and male mice. The E1#3 supernatant (S) sample in **C)** was underloaded. Cropped images are shown in Figure 10.

Table S1. PCR primers

Gene Symbol (Accession Number)	Direction	Sequence	Amplicon size (base pairs)
Cst3 (NM_009976)			527
	Forward	ACGCTCGCATTTGGGTAAAAG	
	Reverse	TCAGCCCTTAGGCATTTTTGC	
	Forward	CATGGCAAAACCCTTGTGGC	
	Reverse	GGTTGAACTCGCCATTCCAG	
Cst9 (NM_009979)			581
	Forward	CCTGTGGGAGTGAAGAGCTAAG	
	Reverse	GCACAATGCTGCAAAACAAGG	
Cst11 (NM_030059)			527
	Forward	GACCCAACAAATGAAGTGGGAG	
	Reverse	ATTGATTGCAGGGGGATTGG	
Cst12 (NM_027054)			494
	Forward	TCTCAGTGCCAACTCTGAAGAC	
	Reverse	AAAGAGACCCCAGACTACAGG	
Cst13 (NM_027024)			446
	Forward	TGGCCAGATTCTTACAGACCC	
	Reverse	GCAAGGTGTCCCGAATGCTG	
Cst14/Cstdc1 (NM_030135)			479
	Forward	AAGGACAGTCATGTCGTGGA	
	Reverse	ACAGACCTTGGGCTACGGA	
Cstdc2 (NM_029960)			470
	Forward	ATCATATTGTCGCCAGCGG	
	Reverse	GGACTGGATCAGTGCTGTAAGT	
Cstl1 (NM_177655)			473
	Forward	TCCGAAGCCATGGAGATGAAG	
	Reverse	CACAGGCATCTGGGTGTCAG	
*Cst8** (cloning primers)			401
	Forward	GCAT**AAGCTT**CATGGCAAAACCCTTGTGGC	
	Reverse	GCAT**GAATTC**GGTTGAACTCGCCATTCCAG	

* Sequences in bold represent HindIII and EcoRI restriction sites.
